# Notes and News

**Published:** 1887-11-26

**Authors:** 


### NOTES AND NEWS.

Collected and Communicated.

The programme of the Parkes Museum contains more than one paper of general interest. Thus Mr. Shirley Murphy, M.R.C.S., will lecture on "Scarlet Fever" on the 15th prox. Dr. Angel Money, assistant physician to the Hospital for Children, on "Hygiene in Childhood" on January 5th ; Miss M. Chreiman on "Physical Culture," on March 15th ; and Mr. A. S. Murray, Keeper of Greek and Roman Antiquities at the British Museum, on "Physical Training of the Greeks and Romans." These lectures are given at five p.m. at the Parkes Museum, to which non-members are admitted on payment of sixpence.

We wonder how many hospital treasurers will follow the example of Mr. G. Herring, of the North-West London Hospital, who proposes making his Jubilee offering by adding one-half to the amount of all sums sent in answer to the appeal which he has issued on behalf of this institution. Formerly those who concerned themselves directly with the management of the hospitals were for the most part men of large means, who gave considerable sums to the support of the institutions with which they were connected. At the present time the governors at large are no doubt much more generally represented on the committees of management than formerly. This change, desirable as it is, has tended, we fear, to the withdrawal or abstention of the wealthier supporters from active participation in the management. At any rate, a comparison of the subscription lists with the office-bearers would enable anyone to judge for himself how far this change has resulted, and it would be refreshing to find that the example set by Mr. Herring was being generally followed by the treasurers of all the voluntary hospitals.

It is stated that a college of medicine for Chinese has been opened at Hong Kong, with Dr. Patrick Manson as Dean, and an able staff.

Coventry is one of the few places where the Hospital Saturday Fund displays a large increase as compared with the amount collected last year. After the payment of all expenses £765 16*s.* 3*d.* was the sum handed to the hospital committee. Last year the total amount was £662.

The annual ball on behalf of the Oswestry Cottage Hospital took place on Tuesday evening at the Victoria Rooms, and was unusually successful. It may be hoped that the recent dispute in connexion with this hospital has been terminated satisfactorily to all parties.

The matron of the Bristol Infirmary is appealing to the public for Christmas gifts for distribution in the children's ward. The Bristol matron is taking time by the forelock, but the approach of the Christmas season should remind all those who have small sums to spare, and a kindly disposition, to remember the hospitals, and especially the children's wards.

The Scottish branch of the Medico-Psychological Association held its quarterly meeting on the 10th inst., in the hall of the Faculty of Physicians in Edinburgh. Dr. Howden, Montrose Royal Asylum, was called to the chair. There were nineteen members present. Several papers of interest were read and discussed, and the meeting generally was animated and successful. The members dined together in the Edinburgh Hotel in the evening. Mr. Frank Lang Collie, M.B., C.M., Clinical Assistant, Perth District Asylum, was elected a member of the Association.

It is understood that plans have been accepted for the erection, in connection with the Montrose Royal Asylum, of a new building to accommodate 100 patients, fifty of each sex. This addition is designed as a hospital for the reception of infirm and recent cases, and, with this in view, special attention has been paid to the provision of adequate floor and cubic space, to ventilation, verandah accommodation, and the plentiful admission of light. The walls of the wards are to be lined with glazed brick, and the building is to be heated throughout by a system either of steam or of hot-water pipes. The structure is mainly of one storey, and of somewhat elegant appearance, and its erection is to be commenced shortly.

Discussing the question of borough sanitation, the *Sheffield Daily Telegraph* says that "a corporation having neither soul to be saved nor an individuality to be kicked, is not peculiarly susceptible to criticism." According to the *Telegraph*, the sanitation of Sheffield is anything but modern. Middens, it appears, are still not unknown in many of the outlying lanes and poorer streets. The corporation, apparently, it is stated, have no appreciation of modern sanitation, being content to do what they are absolutely compelled to do, and nothing more. For intelligence and public spirit the leading inhabitants of Sheffield do not compare favourably with those of some other Yorkshire towns.

Mr. A. Prevost, Treasurer to the University College Hospital, writes to a contemporary : "As there appears to be a very prevalent impression that the University College Hospital has largely benefited from the will of Mr. Richard Quain, the late consulting surgeon to the hospital, I trust that you will allow me to state through your columns that such is not the case. A very large sum was left by Mr. Quain to University College for specific purposes, but by the terms of the will the hospital cannot benefit thereby ; and I fear that if the impression that this charity has received any such legacy is allowed to remain without public contradiction, it may seriously affect the contributions so much needed to enable the hospital to clear off the indebtedness to its bankers and tradesmen, which exceeds £12,000.

The following vacancies are announced this week, the amount of salary in each case being given after the name of the institution :—

**Resident Medical Officers.**

Bolton Infirmary. Junior House Surgeon.—£100 to £150.
Burton-on-Trent Infirmary. House Surgeon.—£130; apartments.
Central London Hospital, Gray's Inn Road. House Surgeon.
Paddington Green Children's Hospital.—£80; rooms.
Stockport Infirmary. Assistant Medical Officer.—£70.
St. Leonard's, Shoreditch, Workhouse Infirmary. Assistant Medical Officer.—£100.
Boscombe Provident Dispensary, Bournemouth. Resident Officer. —£60.
St. George's, Hanover Square, Provident Dispensary. Resident Medical Officer.

**Non-resident Medical Officers.**

Sheffield Medical Officer of Health.—£500.
Bristol Royal Infirmary. Obstetric Physician.
Great Northern Central Hospital. Obstetric Physician. Outpatients.
Portland Urban Sanitary Authority. Medical Officer of Health.
Victoria University, Manchester. Examiners in Surgery, Anatomy, Pathology, Obstetrics, Forensic Medicine.
Westminster Hospital Medical School. Lecturer in Physiology.

**Matrons.**

Beckenham Cottage Hospital. Trained Nurse, to act as Matron. Thirteen beds. State salary required.
Clifton College Sanatorium. Lady Matron. Two years' nursing experience.

**Nurses.**

North Devon Infirmary, Barnstaple. Charge Nurse.—£25.
Denbighshire Infirmary, Denbigh. Head Nurse.—£25; uniform.
Jersey Institution, St. Heliers, Jersey. Several.—£25; uniform.
West London Hospital, Hammersmith Road. Assistant.—£24.
Nottingham and Notts Institute, Nottingham. Several.
Bradford Infirmary, Yorkshire. Night Nurses.—£25.
City of London Hospital for Diseases of the Chest. Nurse.—£20 to £24.
Home for Invalids, Oakfield, Forest Hill. Nurse. State salary required.
West Norfolk and Lynn Hospitals. Night Nurses (2).—£20; uniform.
Nursing Institution, Wimpole Street, W. Trained Nurses. Several.—From £30.
Darlington Fever Hospital. Night Nurse —£27; uniform.
Royal Albert Hospital, Devonport. Nurse to take charge of ward —£25; uniform.
County of Cornwall Trained Nurses' Home. Nurse for Medical and Surgical work.
Nurses' Home, Norwich. Nurse for private work.—£24; uniform.

**Probationers.**

Chelsea Infirmary. Several.

**Laundress and Laundrymaid.**

Seamen's Hospital (late Dreadnought).—£20 and £12 respectively; board and uniform.

---

In consideration of the bequest of £2,000 to the Worthing Infirmary by the late Mrs. Ivory, it has been resolved that the male ward be forthwith set apart, to be for ever called and known as the "Ivory Ward."

---

A new medical relief club on a large scale is suggested for the town of Bridlington. On this subject Dr. Andrew Allison, writing to the *Bridlington Free Press*, says: "I have seldom known gratuitous medical attendance to be largely appreciated either by the rich or parish poor. They are apt to conclude that insufficient remuneration means professional neglect, short visits, or inferior drugs—factors discouraging alike to the doctor, and occasionally fatal to the patient—calculated to alienate sympathy from any hospital, however promising its birth may have been." Those who have had experience of the working of private dispensaries have reason to know that much of what Dr. Allison says is perfectly true. These clubs, conducted on principles which cannot possibly be remunerative if adequate attendance and the best drugs are supplied, do for the most part result in professional neglect, short visits, inferior drugs, danger to the patient, and permanent injury to the professional character of the medical attendant. In many instances they are absolutely demoralising to the doctor, his assistants, and and his patients, and they deserve severe condemnation.

The growth of the Jubilee Fund for the extension of the Aberdeen Royal Infirmary is continuously rapid. The total amount now reaches nearly £26,000.

---

In commemoration of the Jubilee, a new ward has been added to the Beccles Hospital, and the whole building has been painted and renovated throughout.

---

Sir Joseph Fayrer, K.C.S.I., M.D., has accepted a s ea on the Council of The Hospitals Association. New members are now joining in large numbers, thirty ladies and gentlemen having already been elected during the present session.

---

The Barnet Town Hall was crowded to excess on Thursday evening by a highly sympathetic audience, to listen to a concert by Mr. H. Francis Gregg on behalf of the Barnet Cottage Hospital. The net proceeds contributed to the hospital fund amounted to a little over £10.

---

There has been an outbreak of fire at the North-Western Hospital of the Metropolitan Asylums Board. Workmen were engaged in fitting up new hospital huts, and a large quantity of tar that was being heated caught fire, but fortunately the flames, which were quickly extinguished by the workmen and the officials, did not reach the buildings.

---

The next evening meeting of The Hospitals Association will be held on Wednesday, December 14th, at Guy's Hospital, St. Thomas's Street, S.E. (London Bridge), under the presidency of Mr. E. H. Lushington (Treasurer of Guy's), when a paper will be read by Dr. J. C. Steele on the "Registration of Trained Nurses."

---

In reply to a request for the use of the Mansion House for a public meeting on the much-debated question of compulsory vaccination, the Secretary of the London Society for the Abolition of Compulsory Vaccination has received the following reply: "The Mansion House, London, November 12th. Dear Sir,—I am directed by the Lord Mayor to say not only that he must decline the request of your committee to grant the use of the Mansion House for their meeting, but that he is most thoroughly opposed to their sentimental antagonism to a recognised, wise, and humane law.—I am, dear sir, yours very truly, Wm. J. Soulsby."

---

It can never be said that the British nation is indifferent to the rights of property, or even to its privileges; but a story that was told at the Thames Police-court on Thursday, the 17th, will rouse indignation in those who are most ready to justify the landlord's claims. A respectable-looking young woman came to tell of the way in which her landlord attempted to eject her from the room she occupied. She owed him twelve shillings for rent, and to obtain this he had seized her furniture, leaving her and her children in the bare, empty room. On the evening of Wednesday, the 16th, he sent a man to remove the windows and door, in order to compel her to leave the place. She told the messenger that her children were very poorly, and besought him not to expose them to the night air; but in spite of her entreaties, he took out the windows, but left the door, and the family remained till morning in the cold and foggy air of a frosty November night. The magistrate condemned the act as illegal, but legality is a secondary point compared with the brutality of it. A man has a right to receive a rent from the occupant of his property, and to refuse occupancy to any one who cannot pay for it; but in the name of humanity we must protest against a tenant being dismissed from a house without due warrant, or being forced to leave by means of a brutality that is dangerous to life. We should be glad to hear that the woman has brought an action against her landlord, and obtained heavy damages. Such barbarous conduct should not go unpunished.

The foundation-stone of the Children's Jubilee Hospital at Gateshead will be laid on December 2nd. The total cost of the new building will be £6,000, towards which Lord and Lady Northbourne, besides giving the site, have contributed £1,250.

There is a serious outbreak of small-pox at Sheffield—as many as a hundred fresh cases each week. Isolation is impossible, on account of the hospital accommodation being quite inadequate. It has been decided to erect temporary buildings a few miles from Sheffield, to accommodate eighty additional cases.

According to the *Londonderry Sentinel*, the Hospitals Sunday collection in that city produced last year no more than £14, and this, it is stated, was entirely contributed by one Presbyterian congregation. If the other congregations do not blush at such a record there must be something wrong with their vaso-motor systems.

The winter session of the Parkes Museum, 74a, Margaret Street, W., was opened on Thursday, the 17th inst., and the Right Hon. the Earl of Meath read a paper on the importance of open spaces. Lord Brabazon has made this subject all his own, but it is a good sign that other members of the Upper House are using their influence to awaken public attention to a matter which closely concerns the health and welfare of the population of great cities like London.

At a representative meeting of subscribers to the Blackheath and Charlton Cottage Hospital, held last Wednesday, Rev. J. W. Marshall, M.A., in the chair, a resolution was passed *nem. con.* expressing the opinion that: "It is desirable that the necessary steps be taken forthwith to build a new hospital on the Shooter's Hill Road site, so generously offered by the Earl St. Germans," and a Building Committee was appointed to carry this resolution into effect.

Home visitation by the resident medical officers of the Bournemouth Hospital was discussed at some length at the recent meeting of the governors. It was stated that the practice had arisen when both Bournemouth and its hospital were very much smaller than they are now, and that with one house surgeon it seemed to be difficult, if not impossible, that adequate home visitation should be done. There was a strong feeling in favour of the continuance of the practice, and it is not improbable that for this purpose an additional house surgeon may ultimately be appointed.

Working men, it appears, are not to be admitted to life governorships at the Hull Infirmary. In response to a deputation who waited upon the Board of Management, that body has sent the following resolution: "That laws 3 and 4 (constitutional laws) are intended to give a governorship to one person as representative of the bodies named therein; consequently the claim made on behalf of the Hospital Saturday Committee, the Friendly and Trade Societies, and the operatives, is not in accordance with the intentions of the laws.

Messrs. James Dickson and Sons, of Sheffield, have set an example, which, if followed largely, would tend greatly to prevent the spread of infectious diseases. They have decided that during the prevalence of small-pox any workman or workwoman being attacked shall have wages assured until quite well. In the case of their children or other members of their household catching the disease, they shall remain away from the works until all danger of infection is past. During that period their wages shall be paid to them out of a fund to be provided by mutual subscriptions, to take the form of a levy at the rate of two and a-half per cent. of the average earnings of each person, or sixpence in the pound.

At the anniversary meeting of the Salop Infirmary the friends and supporters of the charity accompanied the treasurer (Mr. T. Slaney Eyton) to church. There were present also the Hon. R. C. Herbert, Mr. J. Watson, M.P., Mr. W. A. Sparrow, Major-General Hardy, the Rev. T. J. Rider (vicar of Baschurch), Dr. Harris, and others. Plates were held at the door by Miss Slaney Eyton, Lord Kenyon, Mrs. Corrie, and Mrs. Edward Herbert. The total amount collected was £169 2s. 8d.

Presiding at the opening meeting of the Society of Arts the other evening, Sir Douglas Galton mentioned that the City and Guilds of London Institute and the School Board for London were co-operating in an experiment of technical training in six of the metropolitan elementary schools. Lessons are to be given in carpentry, and the co-operation of teachers seems assured by the intelligence that already 80 of them have gone through a course of training in the Finsbury Technical School, to qualify themselves to assist in the instruction to be given in this new and useful branch of education.

In razing the walls of the old Royal Infirmary at Liverpool to the ground, to make way for the new building which is to replace it, the foundation-stone which was laid in 1821 has been discovered. In the centre of the stone was a cavity containing coins of the realm, and covering it was a brass plate recording the incident. The memorial is of much interest as affecting Liverpool families of a past generation —many of whom in their descendants still take an active share in the management of the hospital. The stone was laid in the reign of George IV. by the then President, Edward Baron Stanley, whose descendant, the present Earl of Derby, has just laid the foundation-stone of the new building.

"The general practitioner," says the *Evening Standard*, "has fallen on bad times. A variety of circumstances conspire to undermine his position. Dispensaries have sprung up like mushrooms throughout the country; under careful nurturing the out-patient system of the hospitals is manifesting an active growth; the army of 'specialists' is daily recruited; trade is still depressed; patent medicines and other nostrums are becoming as plentiful as blackberries in September; the profession is overcrowded; chemists are contracting the habit of prescribing, and medical literature is finding its way even to Mr. Smith's bookstalls. Not that these circumstances are all equally inimical to the interests of general practitioners. The influence of some is of course but slight, though it is a striking testimony to the acuteness of the distress that in the controversy on the subject which now agitates the medical world, stress is laid even upon these comparatively insignificant considerations. Speaking broadly, the general practitioner is suffering in four ways. In the first place, his poor patients have left him. They have decamped in a body to the dispensaries and sick clubs and to the out-patient department at the hospitals. Then, too, the critical cases have passed beyond his ken. The 'specialist' and the 'eminent physician' grow fat upon them, and the loss to the general practitioners has involved the removal of many a good mare from their stables. Thirdly, what may be classed as petty and imagined disorders have ceased to yield anything like their proper quota. Again, men of established reputation have been confronted with the alternative either of reducing their fees or losing their patients. The overcrowded state of the profession is said to be a primary cause of the reduction that has taken place in the fees of the general practitioner. Statistics would seem to bear out the contention, for while the average number of deaths of registered medical men for the past ten years has only been 585, the average annual number of new names added to the register has been no less than 1,135, an advance that is certainly more than commensurate with the increase of the population."

